# Extreme Uterine and Rectal Prolapse in a 31-Year-Old Patient: A Case Report

**DOI:** 10.3390/jcm14051484

**Published:** 2025-02-23

**Authors:** Marcin Jozwik, Maria Derkaczew, Joanna Wojtkiewicz, Burghard Abendstein, Maciej Jozwik

**Affiliations:** 1 Department of Gynecology and Obstetrics, Collegium Medicum, University of Warmia and Mazury, 10-045 Olsztyn, Poland; 2 Students’ Scientific Club of Pathophysiologists, Department of Human Physiology and Pathophysiology, School of Medicine, University of Warmia and Mazury, 10-082 Olsztyn, Poland; 3 Department of Human Physiology and Pathophysiology, School of Medicine, Collegium Medicum, University of Warmia and Mazury, 10-082 Olsztyn, Poland; 4 Department of Gynaecology, Academic Teaching Hospital Feldkirch, 6800 Feldkirch, Austria; 5 Department of Gynecology and Gynecologic Oncology, Medical University of Białystok, 15-276 Białystok, Poland; maciej.jozwik@umb.edu.pl

**Keywords:** pelvic organ prolapse, uterine prolapse, rectal prolapse, rectocele, total repair

## Abstract

**Background:** Pelvic organ prolapse (POP) is a common disorder among postmenopausal women but is rare in very young patients. It can affect various compartments of the pelvic floor. In severe forms, vaginal/uterine and rectal prolapse can occur concurrently. **Methods:** The aim of this report is to present a rare case of a young patient with an extreme postpartum uterine and rectal prolapse and our stepwise surgical approach to achieve complete repair while preserving the ability to carry future pregnancies. **Results:** A 31-year-old patient was admitted with extreme postpartum uterine and rectal prolapse. She underwent three separate surgeries to regain full anatomic reconstruction. Initially, laparoscopic lateral suspension (LLS) according to Dubuisson’s technique was performed in 2017. A combined vaginal-laparoscopic repair followed again in 2017 and included extensive posterior vaginal and perineal repair with absorbable mesh (SeraSynth) attached to the sacrouterine ligaments and laparoscopic hysterosacropexy (HySa) with a non-absorbable PVDF DynaMesh-CESA implant. Finally, in 2019, the DynaMesh-CESA implant was replaced with a T-shaped non-absorbable Albis Posterior Mesh for rectal prolapse, fixed bilaterally to the sacral bone at the S3 level. Additionally, the Dubuisson suspension was adjusted using Noé’s pectopexy for the implant’s reattachment to the pectineal ligaments. **Conclusions:** Severe uterine and rectal prolapse in young patients is rare and demands a tailored approach. Uterus-preserving surgery should be the priority. In the present case, a resorbable posterior mesh failed in rectal prolapse repair, while a combined rectal prolapse repair and hysteropexy with a non-resorbable posterior mesh proved effective.

## 1. Introduction

Pelvic organ prolapse (POP) is a widespread condition that can impact any part of the pelvic floor. The prolapse is defined as a descent of the urethra, bladder, vagina, small intestine, and/or rectum [1]. Being mostly a delayed consequence of vaginal deliveries, POP primarily affects postmenopausal women due to the weakening of connective tissue deprived of estrogen stimulation. The exact incidence in younger women is unclear, as many individuals with POP do not seek treatment. It has been reported that the occurrence of POP in women under 40 years of age is 1.6% [2]. Common symptoms for POP include a noticeable bulge, external protrusion, a feeling of fullness in the vagina, discomfort or pain in the pelvic region, lower back pain, dyspareunia, urination problems such as frequent micturition, a feeling of incomplete bladder emptying, urinary incontinence, bowel movement difficulties like constipation, and dragging sensation in the pelvis. A significant rectocele is defined as the protrusion of the anterior rectal wall, presenting as a bulge, visible through the posterior vaginal wall due to the weakening or loss of the rectovaginal septum.

POP prevalence estimates range from 2.9% to 11.4% when using questionnaires but increase to over 50% when using physical examination [3]. One of the widely used scales to assess the severity of POP is the Pelvic Organ Prolapse Quantification System (POP-Q) [4]. It is a five-stage scale from zero to four, which enables the assessment and clinical comparison of how far the prolapse has descended. The guidelines for using the POP-Q scale are presented in Table 1.

In about 40% of patients, more than one compartment is affected, though rectal prolapse is uncommon and mostly affects elderly individuals [5]. In such cases, multiple access surgery may be indicated. It has been highlighted that changes at the molecular level in connective tissue contribute significantly to the onset of POP. The pelvic floor’s connective tissue, which includes ligaments and fasciae, is mainly composed of extracellular matrix components such as collagen, elastin, and other fibrous structures. Major risk factors for POP include advanced age, lifestyle choices, high number of pregnancies and childbirths, obesity, and conditions associated with elevated intra-abdominal pressure. Other contributing elements include the mode of delivery, hormonal changes related to estrogens, a history of pelvic surgeries, and genetic factors [6,7]. A weakened pelvic floor and reduced structural support, along with the individual presence of risk factors, can lead to the simultaneous prolapse of nearby pelvic organs, including the rectum [8].

We present a case report of a patient of reproductive age who was admitted to our institution due to advanced postpartum uterine and rectal prolapse for combined vaginal/laparoscopic reconstruction, with the goal of preserving her ability to bear children.

## 2. Case Report

A 31-year-old woman (G2, P2) presented to our outpatient department in October 2017 with a recurrence of total uterine and rectal prolapse. She had a history of a vaginal delivery (at 40 weeks gestation, with oxytocin stimulation and episiotomy) in 2006 and a Cesarean section in 2007 (at 38 weeks gestation, PROM, oxytocin stimulation), both with no complications. Her first child was a male weighing 3800 g and her second child was a female weighing 3600 g. The prolapse initially appeared in 2016, i.e., about 10 years after her first delivery. Of note, the patient had a longstanding history of Crohn’s disease since her childhood, with symptoms limited to abdominal discomfort and intermittent loose stools, but without constipation.

**Figure 1:**
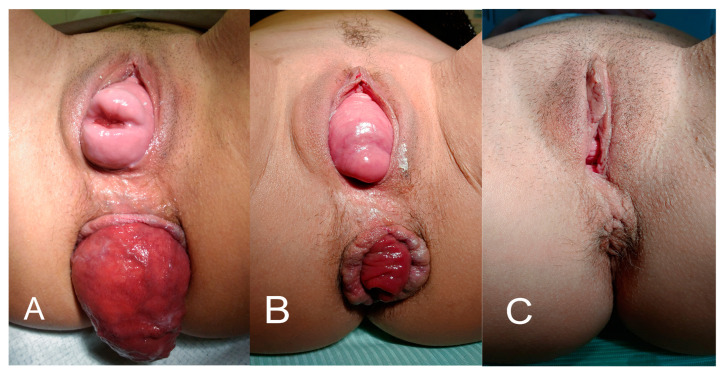
(**A**) Despite the first surgery in February 2017, a total uterine prolapse and rectal prolapse were pronounced at the patient’s admission to our institution in November 2017. (**B**) Partial improvement with a visible rectocele relapse after a vaginal resorbable mesh placement in the posterior compartment combined with a laparoscopic bilateral hysterosacropexy with a non-resorbable DynaMesh-CESA implant (performed November 2017) at a checkup in December 2018. (**C**) The final outcome: complete improvement after laparoscopic implantation of a non-resorbable mesh in the posterior compartment with fixation of this implant to the cervix and sacral bone, combined with the bilateral correction of the tension of lateral suspension mesh arms (performed in April 2019). Results stable as of December 2024.

**Figure 2:**
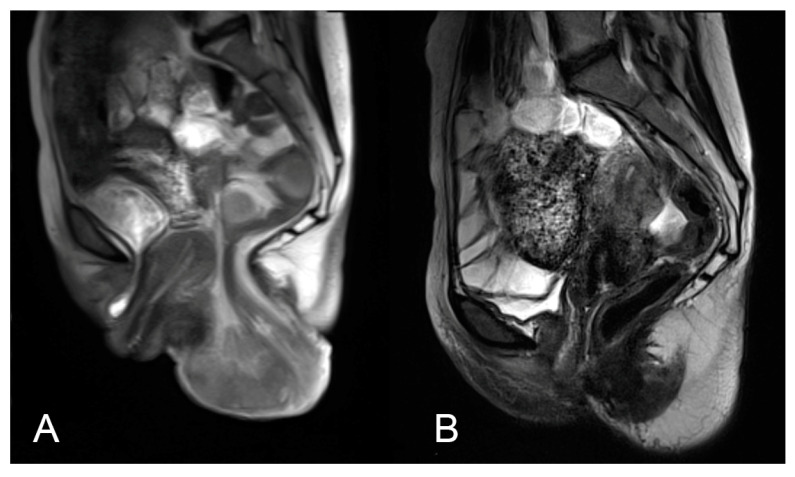
MRI of pelvic organs in sagittal view. (**A**) Total uterine and rectal prolapse at the time of admission in November 2017. (**B**) Final outcome after treatment in April 2018. This anatomical picture remains stable as of December 2024.

Her first surgery for the prolapse had been performed in another hospital in February 2017. It consisted of the Dubuisson’s laparoscopic lateral suspension (LLS) of the uterus using a T-shaped implant [9]. In 2018, the patient was diagnosed with Addison’s disease and started treatment with hydrocortisone. She had a sedentary occupation as a teacher, was a non-smoker, and had a normal body mass index (57 kg, 170 cm). No other risk factors were identified in her history besides her hydrocortisone intake; however, as the prolapse appeared before this treatment, it was clearly of postpartum origin. Upon admission to our institution in October 2017, the patient reported the recurrence of symptoms, including low abdominal pain, dyspareunia, and defecation problems (loose stools). A clinical examination confirmed a uterine prolapse of POP-Q stage 4, a cystocele with a lateral defect of POP-Q stage 2, a rectocele of POP-Q stage 2, as well as a complete rectal prolapse (Figure 1A). Magnetic resonance imaging (MRI) confirmed a total uterine and rectal prolapse (Figure 2A). The patient sought treatment to maintain her fertility. 

In November 2017, we performed a combined vaginal-laparoscopic pelvic floor repair surgery with uterine preservation, which included: (i) extensive posterior vaginal and perineal repair with reinforcement of the rectovaginal septum using a fully absorbable mesh (SeraSynth, 100% PDS by Serag-Wiessner, Naila, Germany) with laparoscopic attachment of the mesh to the sacrouterine ligaments; and (ii) laparoscopic hysterosacropexy (HySa) using a non-resorbable PVDF DynaMesh-CESA implant (FEG, Dahlhausen, Germany). The absorbable mesh was chosen to prevent erosion. Both the surgery and postoperative period were uneventful.

A year later (December 2018) the patient returned to our department with another recurrence, but this time her defect was pronounced in the posterior compartment, with a rectocele POP-Q stage 3 and a recurrent rectal prolapse (Figure 1B).

**Figure 3:**
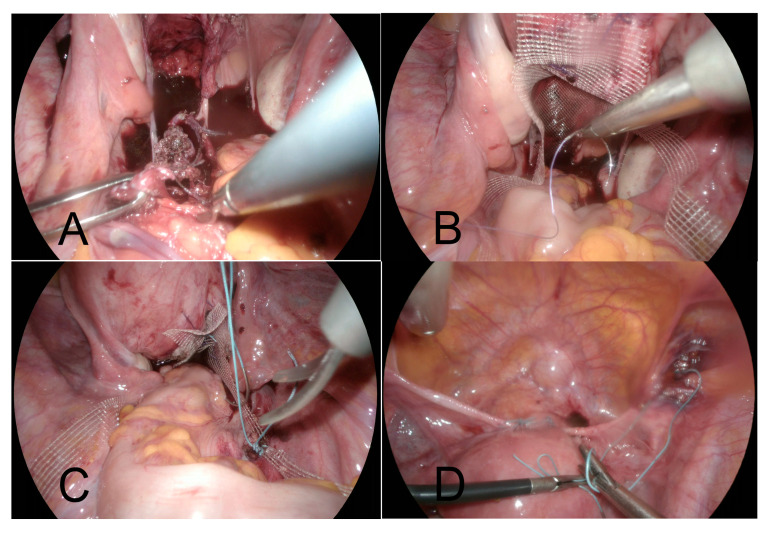
Laparoscopic steps of the final (third) surgical procedure (April 2019). (**A**) Removal of the DynaMesh-CESA implant. (**B**) Placement of the Albis Posterior Mesh deep into the rectovaginal space. (**C**) Fixation of the Albis Posterior Mesh to the posterior cervical wall and bilaterally to the sacral bone. (**D**) Reattachment of the already present Dubuisson’s implant to the pectineal ligaments according to the uterine pectopexy technique described by Noé et al.

During the subsequent laparoscopic surgery (April 2019), (i) the DynaMesh-CESA implant was removed (Figure 3A), (ii) a non-resorbable mesh (Albis Posterior Mesh by Albis Mazur Sp. z o.o., Kalisz, Poland) was placed between the posterior vaginal wall and the anterior rectal wall, covering the full plane from the levator ani muscles up to the posterior cervical wall (Figure 3B) with bilateral sacrofixation of this ‘T’-shaped mesh at the S3 level (Figure 3C). At the same time, (iii) the tension of the Dubuisson’s lateral suspension implant was trimmed according to Noé’s uterine pectopexy technique [10], with the additional reattachment of the present implant to the pectineal ligaments (Figure 3D). Again, the procedure and postoperative period turned out uneventful. Figure 2B depicts in MRI the effects of the third operation. To prevent further pelvic floor injuries, the patient was advised to undergo Cesarean sections for any future pregnancies.

Since her last surgery in April 2019, the patient has been doing fine with no complains related to pelvic organ anatomy or function. Dyspareunia and lower abdominal pain have completely resolved and micturition is normal. Her Crohn’s disease has been treated with azathioprine since 2022, and loose stools appear only occasionally. She continued her treatment for Addison’s disease from 2018 until 2023, when she spontaneously stopped taking hydrocortisone, with no reported adverse effects. Also, she chose not to become pregnant again. At her checkup in December 2024, the anatomical positioning of her pelvic organs remained fully stable.

## 3. Discussion

Total uterine prolapse in a patient in her early thirties is a rare condition, as POP is typically associated with postmenopausal women or those who underwent multiple vaginal deliveries over an extended period. The presence of both vaginal and anal prolapse in such a young patient adds another layer of complexity to the case, making it imperative to consider long-term anatomical and functional outcomes. In women of this age, special attention should be paid not to compromise her fertility by prolapse treatment. For many years, uterine removal was performed during prolapse repair surgeries. However, more recent studies have shown similar results when prolapse repair without hysterectomy follows. This new approach has been shown to reduce complications associated with concurrent hysterectomy during prolapse repair. For instance, hysteropexy alone results in shorter operative times, less blood loss, and fewer perioperative complications than surgeries involving hysterectomy [11,12]. Moreover, the uterine muscle, and the uterine cervix in particular, has been identified as a stable site for the implant attachment during suspension. The choice between uterine preservation and removal should be based on the individual patient’s factors, including age, medical history, symptoms, personal preferences and reproductive plans in particular [13].

Vaginal delivery is considered a significant etiological factor in the development of vaginal prolapse [7]. The process of childbirth, particularly vaginal and forceps delivery, places considerable strain on the pelvic floor muscles and surrounding connective tissues, which are responsible for supporting the vaginal walls and other pelvic organs. Repeated vaginal deliveries, especially in case of prolonged labor, large birth weights, or the use of forceps, can further weaken these structures. Such a weakening leads to a reduced ability of the pelvic floor to provide adequate support, making the vaginal walls more prone to prolapse. Over time, the cumulative effects of childbirth, combined with other risk factors such as progressing age, hormonal changes and obesity, may increase the likelihood of vaginal prolapse, contributing to its development in women who have experienced one or more vaginal deliveries [14,15]. In our case, the patient’s first childbirth was a vaginal delivery, consistent with many reports on the most strongly associated risk factors for the development of POP.

Generally, a single surgical plan including more than one compartment is sufficient to regain anatomical repair. In the case of our patient, three successive surgical attempts were necessary to finally manage the defects. In our opinion, this was due to a combination of multiple contributing factors, such as defects at all three pelvic-floor compartment levels, including rectal descent, the known detrimental effects of hydrocortisone treatment for Addison’s disease on connective tissue, and, in particular, the patient’s individual susceptibility, or connective tissue incompetence. Alterations in the elastic fibers within the dermis, as observed in patients with rectal prolapse, suggest a potential connective tissue disorder contributing to the defect. These changes can lead to the weakened support of structures within the pelvic floor, contributing to the development of prolapse. In our patient’s case, a history of hydrocortisone intake further complicated her condition, as the medication is known to influence connective tissue integrity by decreasing collagen synthesis and increasing the breakdown of collagen and elastin [16,17]. On the other hand, women face an increased likelihood of POP recurrence within the first two years following surgical intervention, irrespective of the number or combination of compartments addressed during the initial procedure [18]. In our patient, the first symptoms of the disease were observed in 2016, and each recurrence occurred approximately one year after the surgery.

The qualification for pelvic floor reconstruction surgery is determined based on the severity of prolapse involving the reproductive organs, bladder, or rectum, as well as the symptoms reported by the patient. Various surgical approaches have been employed, including vaginal and abdominal access, with the latter encompassing both laparoscopic and open laparotomy techniques [19]. Weakened tissues and prolapsed organs can be supported and suspended with the use of implanted materials: biological (grafts) or synthetic (meshes). Autologous grafts are usually made from the rectus sheath or fascia lata. Biological grafts were used more commonly in the 1990s. However, over time their application was abandonned in favor of synthetic materials due to the risk of infection transmission and poor long-term outcomes. Synthetic meshes can be divided into absorbable or non-absorbable materials. Absorbable meshes are usually made of polyglactin or other polymers and are replaced over time with connective tissue rich in collagen [20]. These meshes are often used in cases where long-term foreign material retention is undesirable or may produce erosion. However, since they degrade, they may not provide sufficient for long-term reinforcement, increasing the risk of prolapse recurrence [21].

There are a variety of techniques used for prolapse treatment. The Dubuisson technique, also named LLS, is a minimally invasive laparoscopic surgical approach used to treat POP, particularly apical prolapse involving the uterus, cervix, or vaginal vault. This technique relies on the use of a synthetic mesh to create lateral support for the pelvic organs. Since its introduction in 1998, the Dubuisson technique has gained attention for its high success rate and reduced postoperative complications, with infrequent long-term mesh-related issues. This method is particularly valued for its tailored approach, allowing to preserve the uterus, and is becoming increasingly standardized in apical prolapse repairs. During the procedure the anterior vaginal wall, along with the cervix, are being elevated and secured using a mesh attached to the fascia of the external oblique abdominal muscle [19,22,23]. Hysterosacropexy has become a standard surgical procedure used to address uterine prolapse. It is often the preferred method for the treatment of uterine prolapse in patients who wish to preserve their uterus. This was the case with our patient who desired to maintain her fertility [24,25]. Hysterosacropexy or colposacropexy involve suspending the prolapsed organ to the sacrum, with the optimal fixation site being at the S2–S3 level. This location is preferred over the promontory as it preserves the natural alignment of the vagina [19,26]. The literature supports the possibility of pregnancy following hysterosacropexy. Such reports indicate that patients remain asymptomatic during pregnancy, with no signs of prolapse recurrence, and such pregnancies were concluded with planned Cesarean sections [27,28,29]. Posterior mesh repair is a technique that involves placing a mesh between the posterior vaginal wall and the anterior rectal wall, optimally anchoring deep at the levator ani level and with the sacrospinal fixation of the posterior mesh arms. Here, the goal is to restore the anatomical position of the rectum and vagina. A key feature of this method is to provide the long-term support, making a non-absorbable mesh the preferred option. In our patient, the posterior mesh was additionally fixed to the posterior cervical wall and secured at a high sacral level for maximum reinforcement of the posterior compartment structures, as suggested [30,31].

Additionally, the tension of the mesh already present after the previous Dubuisson operation was corrected according to Noé’s pectopexy technique. Pectopexy is an advanced minimally invasive surgical technique for the correction of the middle compartment that aims to suspend the uterus, cervix or vaginal vault by bilateral attaching it to the pectineal ligaments. Pectopexy provides a strong attachment to the pelvic brim and thus stable support to the middle compartment [10,32].

In retrospect, it seems that all the techniques implemented in our patient were appropriate, as each of them represents a proven effective treatment based on others and our experience. We attribute the repeated need for surgery as a reflection of our efforts to apply the least invasive surgical approach possible to avoid mesh complications, particularly dyspareunia and erosion, while preserving the patient’s ability to bear children in the future.

## 4. Conclusions

Extreme uterine and rectal prolapse in young patients is rare but requires a dedicated, individualized approach. Surgery with uterine preservation should always be considered. In our case, the application of a resorbable posterior mesh proved ineffective for rectal prolapse repair. A simultaneous hysteropexy and rectal prolapse repair with a posterior non-resorbable mesh implant was found to be a feasible and effective treatment of advanced defects. In rectal prolapse, sacral fixation of the posterior mesh seems to be an anatomically better option than sacrospinous posterior mesh fixation.

## Figures and Tables

**Table 1:** Pelvic Organ Prolapse Quantification System.

Pelvic Organ Prolapse Quantification (POP-Q) Exam	
Stage 0	No prolapse, Aa, Ap, Ba, Bp are −3 cm and C or D is between −TVL and –(TVL − 2) cm
Stage 1	Criteria for Stage 0 are not met and the leading point of prolapse is 1 cm above the level of the hymen (quantification value is <−1 cm)
Stage 2	The leading point of prolapse is less than 1 cm proximal or distal to the level of the hymen (quantification value is between ≥−1 and ≤+1 cm)
Stage 3	The leading point of prolapse is >1 cm from the hymen plane but no further than 2 cm less than TVL (quantification value is between >+1 cm and <+(TVL − 2) cm
Stage 4	The leading point of prolapse is ≥+(TVL − 2) cm, complete eversion of the total length of the lower genital tract

Aa—anterior vaginal wall, 3 cm proximal to the hymen; Ap—posterior vaginal wall, 3 cm proximal to the hymen; Ba—most distal position of the remaining upper anterior vaginal wall; Bp—most distal position of the remaining upper posterior vaginal wall; C—most distal edge of cervix or vaginal cuff scar; D—posterior fornix; TVL—total vaginal length—depth of the vagina when point D or C is brought back to its normal position.

## Data Availability

The original contributions presented in the study are included in the article. Further inquiries can be directed to the corresponding authors.

## Abbreviations

LLS	Laparoscopic Lateral Suspension
POP	Pelvic Organ Prolapse
POP-Q	Pelvic Organ Prolapse Quantification
PROM	Premature Rupture of Membranes
TVL	Total Vaginal Length
